# Supplementary material for: Correlative voltage imaging and cryo-electron tomography bridge neuronal activity and molecular structure
Source: Nat Commun. 2025 Oct 23;16:9378. doi: 10.1038/s41467-025-64431-w (PMC12550085; doi:10.1038/s41467-025-64431-w)
Supplement: Supplementary file 1 — Supplementary Information [file 41467_2025_64431_MOESM1_ESM.pdf]

SUPPLEMENTARY INFORMATION

Correlative Voltage Imaging and Cryo-Electron Tomography Bridge Neuronal Activity and Molecular Structure

Mingyu Jung, Gwanho Ko, Dongsung Lim, Seonghoon Kim, Sojeong Kim, Young Joon Kim, Myunghwan Choi, Soung-Hun Roh

LIST OF SUPPLEMENTAL INFORMATION

Supplementary Fig. 1. Three-dimensional analysis of electric field simulation

Supplementary Fig. 2. Voltage imaging of HEK293T cell with electric field stimulation

Supplementary Fig. 3. Neuronal responses according to the location on the grids across four different grids.

Supplementary Fig. 4. Overall workflow of subtomogram analysis of ribosomes

Supplementary Fig. 5. FSC curve of the consensus map and each conformation

Supplementary Fig. 6. Statistical analysis of each conformational state of polysomes in different clusters

Supplementary Table. 1. Cryo-EM data collection, refinement and validation statistics

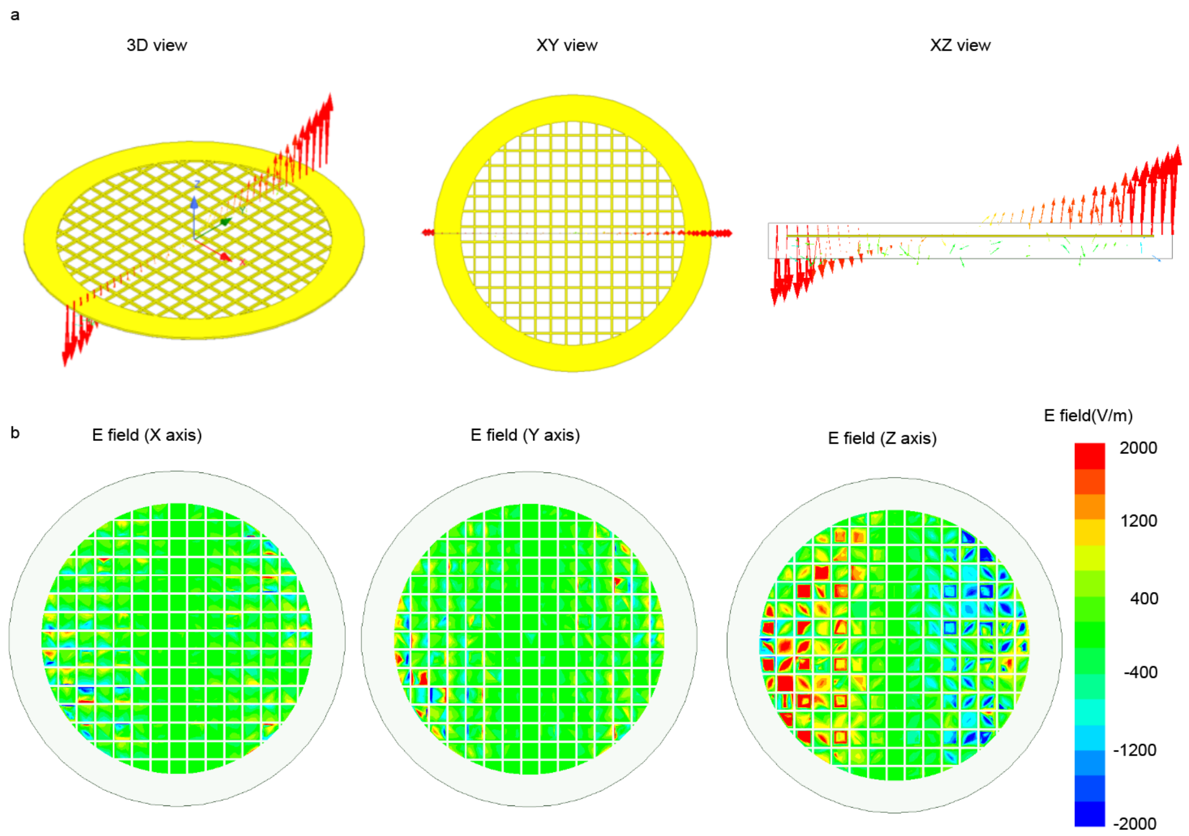

Supplementary Fig. 1. Three-dimensional analysis of electric field simulation.
a. Vector of the electric field shown in various views. Electrodes are positioned at x-axis. **b.** Magnitude of applied electric field along x, y and z axis.

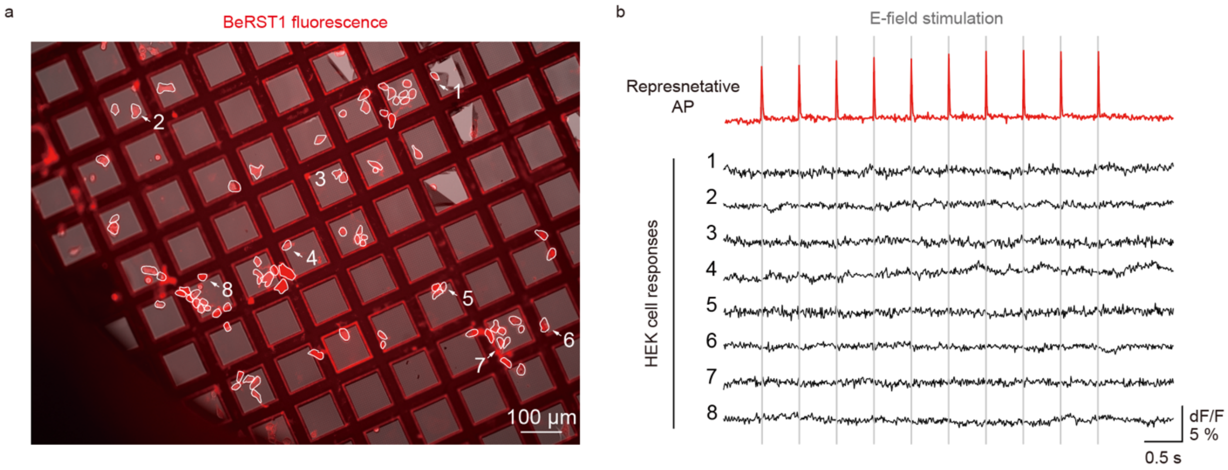

Supplementary Fig. 2. Voltage imaging of HEK293T cell with electric field stimulation
a. On-grid voltage imaging of HEK293T cells during electric field stimulation. HEK293T cells on a grid were stained with the dye BeRST1 (red). **b.** Representative $\Delta F/F$ fluorescence intensity changes according to time from annotated HEK293T cells in **a**. The shaded line indicates the electric field stimulation time.

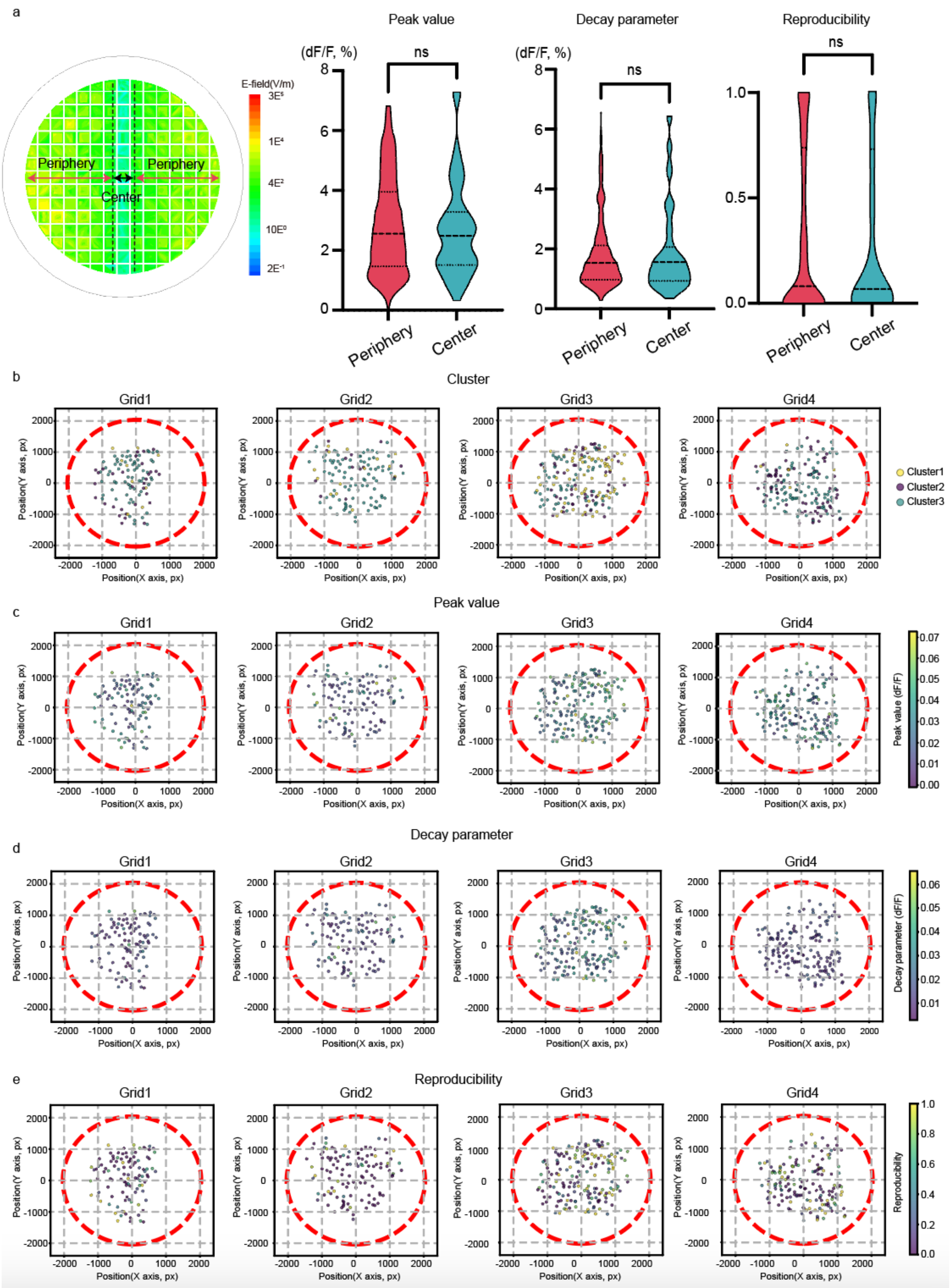

Supplementary Fig. 3. Neuronal responses according to the location on the grids across four different grids.

a. Comparison of neuronal responses in each parameter between neurons located at the center and periphery of the grids is shown as a violin plot (the number of neurons; periphery: 499, center:69). Dashed lines indicate quartile points. Unpaired two-sided t-test was performed to assess statistical significance. ns indicated statistically non significant **b-e.** Spatial mapping of neuronal responses **b.**

Cluster, **c**. Peak value, **d.** Decay parameter **e.** Reproducibility is represented. Source data are provided in the Source Data file.

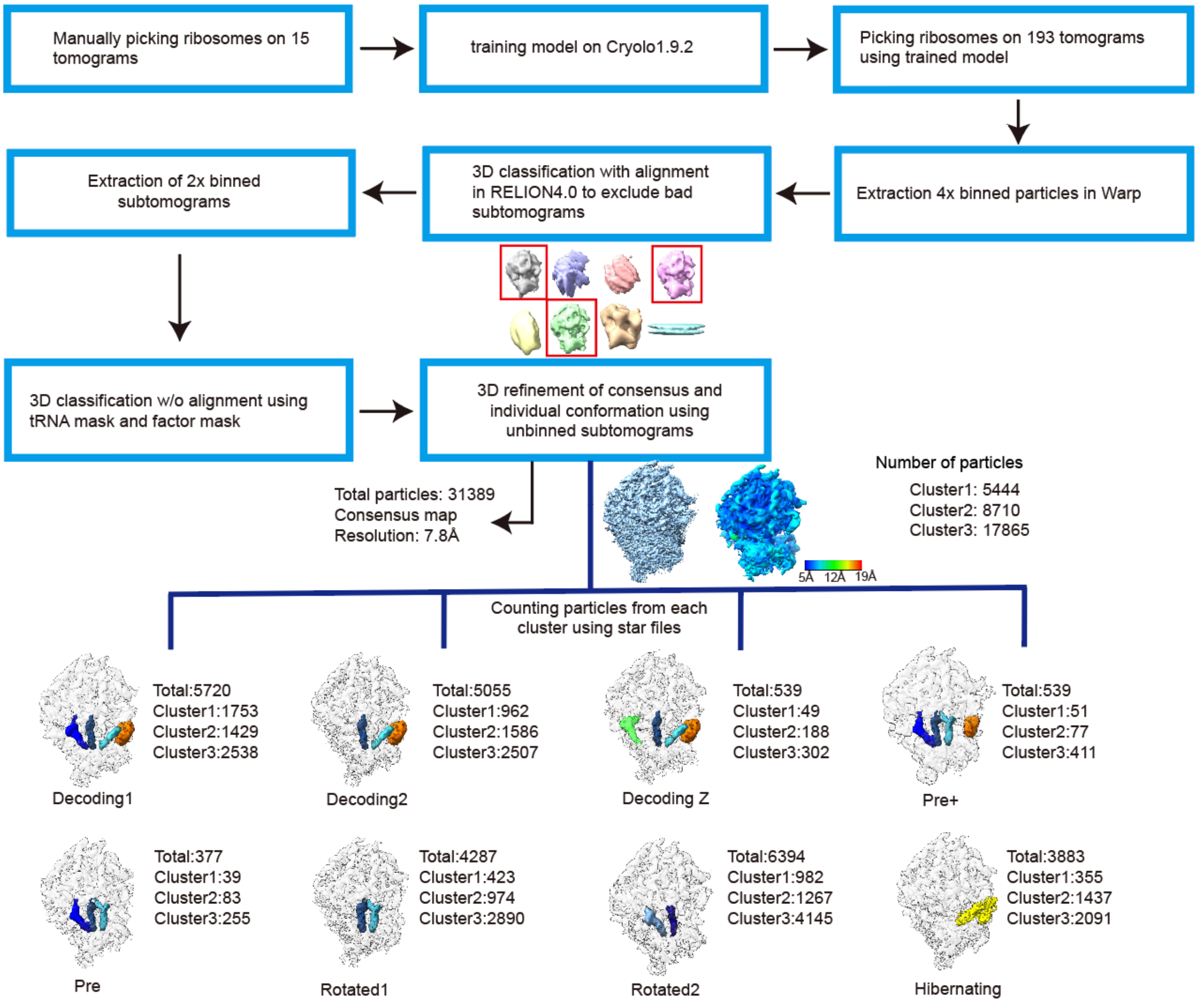

Supplementary Fig. 4. Schematic diagrams of the overall workflow.
Deep learning-based picked subtomograms were extracted and filtered using iterative 3D classification with alignments. Re-extracted 2x binned subtomograms and masks, including the E (Exit), P (Peptidyl), and A (Aminoacyl) sites, along with elongation factors, were used to classify 8 different ribosome classes. Using unbinned subtomograms, final structures were acquired. Information from star files was utilized to count the number of particles in each state from different clusters, which were then used for statistical analysis.

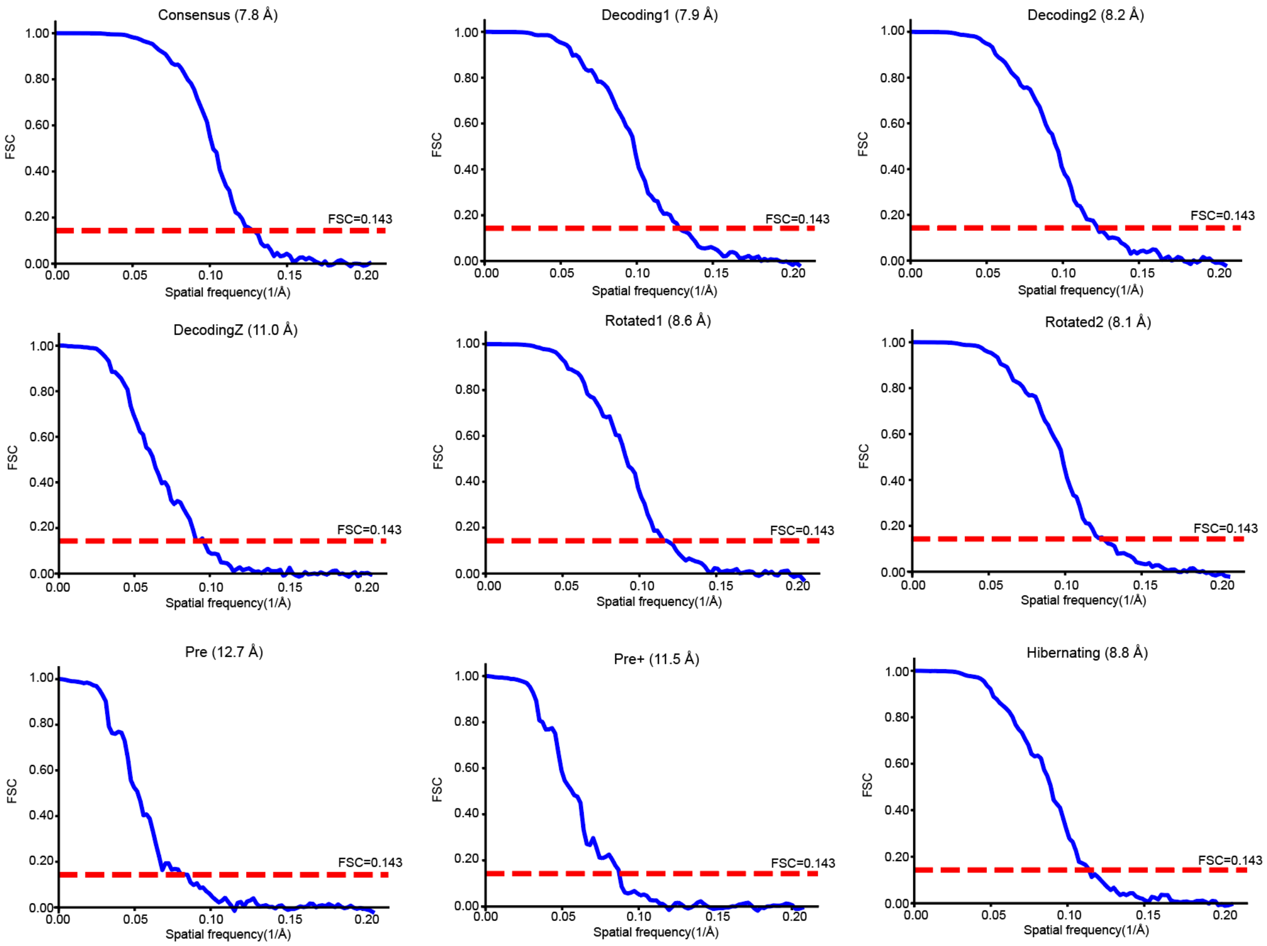

Supplementary Fig. 5. FSC curve of the consensus map and each conformation

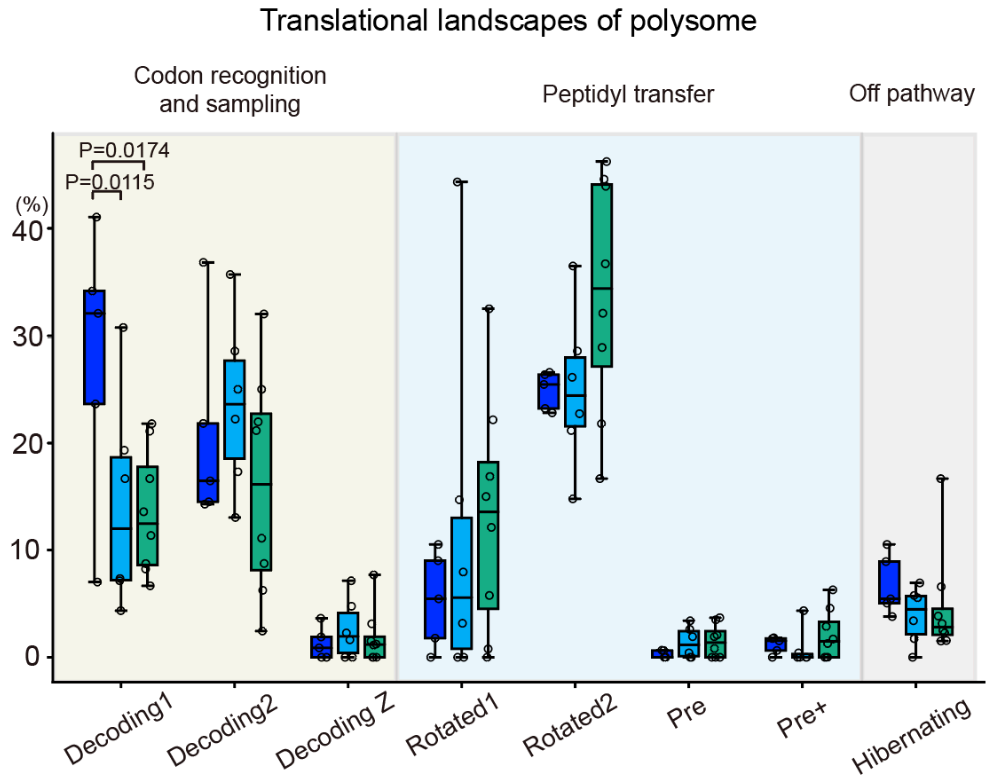

Supplementary Fig. 6. Statistical analysis of each conformational state of polysomes in different clusters

The conformational states of the polysomes where the distance between ribosomes was less than 9 nm were presented as box and whisker plots (bold line: median, box: 25~75 percentile, whiskers: min to max). One-way ANOVA followed by Tukey's post hoc test for multiple comparisons was performed to assess statistical significance, using the portion of individual neurons as the unit of analysis (Cluster 1: n = 5 neurons; Cluster 2: n = 6 neurons; Cluster 3: n = 8 neurons). For statistical testing only, each data point was weighted by the total number of ribosomes in the corresponding neuron. Source data are provided as a Source Data file.

Supplementary Table. 1. Cryo-EM data collection, refinement and validation statistics

	Consensus	Decoding1	Decoding2	DecodingZ	Hibernating
Magnification	53,000				
Voltage(kV)	300				
Defocus (μm)	-3 ~ -4.5				
Imaging pixel size (Å)	2.42				
Processing pixel size (Å)	2.42	4.84			
Symmetry	C1				
Particle images(no.)		5,720	5,055	539	3,883
Map resolution(Å) FSC threshold 0.143	7.8	7.9	8.2	11.0	8.8
EMDB ID	61,318	61,633	61,634	61,635	61,636

	Pre with factor	Pre without factor	Rotated1	Rotated2
Magnification	53,000			
Voltage(kV)	300			
Defocus (μm)	-3 ~ -4.5			
Imaging pixel size (Å)	2.42			
Processing pixel size (Å)	4.84			
Symmetry	C1			
Particle images(no.)	539	377	4,287	6,394
Map resolution(Å) FSC threshold 0.143	11.5	12.7	8.6	8.1
EMDB ID	61,637	61,638	61,639	61,640
