# Supplementary material for: Correlative voltage imaging and cryo-electron tomography bridge neuronal activity and molecular structure
Source: Nat Commun. 2025 Oct 23;16:9378. doi: 10.1038/s41467-025-64431-w (PMC12550085; doi:10.1038/s41467-025-64431-w)
Supplement: Supplementary file 2 — Reporting Summary [file 41467_2025_64431_MOESM2_ESM.pdf]

nature portfolio

Corresponding author(s): Soung-Hun Roh, Myunghwan Choi

Last updated by author(s): Sep 1, 2025

Reporting Summary

Nature Portfolio wishes to improve the reproducibility of the work that we publish. This form provides structure for consistency and transparency in reporting. For further information on Nature Portfolio policies, see our Editorial Policies and the Editorial Policy Checklist.

Statistics

For all statistical analyses, confirm that the following items are present in the figure legend, table legend, main text, or Methods section.

n/a	Confirmed	
☐	☒	The exact sample size (*n*) for each experimental group/condition, given as a discrete number and unit of measurement
☐	☒	A statement on whether measurements were taken from distinct samples or whether the same sample was measured repeatedly
☐	☒	The statistical test(s) used AND whether they are one- or two-sided<br>*Only common tests should be described solely by name; describe more complex techniques in the Methods section.*
☐	☒	A description of all covariates tested
☐	☒	A description of any assumptions or corrections, such as tests of normality and adjustment for multiple comparisons
☐	☒	A full description of the statistical parameters including central tendency (e.g. means) or other basic estimates (e.g. regression coefficient) AND variation (e.g. standard deviation) or associated estimates of uncertainty (e.g. confidence intervals)
☐	☒	For null hypothesis testing, the test statistic (e.g. *F*, *t*, *r*) with confidence intervals, effect sizes, degrees of freedom and *P* value noted<br>*Give P values as exact values whenever suitable.*
☒	☐	For Bayesian analysis, information on the choice of priors and Markov chain Monte Carlo settings
☐	☒	For hierarchical and complex designs, identification of the appropriate level for tests and full reporting of outcomes
☒	☐	Estimates of effect sizes (e.g. Cohen's *d*, Pearson's *r*), indicating how they were calculated

Our web collection on statistics for biologists contains articles on many of the points above.

Software and code

Policy information about availability of computer code

Data collection	We used NIS-Element AR 5.21.03 for voltage imaging data collection.<br>We used Tomography 5 (Thermofisher) for data collection.
Data analysis	VAST Lite 1.4.1 for neuronal soma segmentation, Warp/M 1.10, Relion 4.0 for subtomogram averaging, IMOD 4.11 and ChimeraX v1.3 for visualization. Prism 10 for visualization of graph https://github.com/builab/subtomo2Chimera for mapping subtomogram on tomoram, https://github.com/teamtomo/membrain-seg for segmentation of tomograms, https://github.com/mvanevic/polysome_mef for polysome analysis. https://github.com/Dangallll/CoVET for voltage imaging data analysis.

For manuscripts utilizing custom algorithms or software that are central to the research but not yet described in published literature, software must be made available to editors and reviewers. We strongly encourage code deposition in a community repository (e.g. GitHub). See the Nature Portfolio guidelines for submitting code & software for further information.

Data

Policy information about availability of data

All manuscripts must include a data availability statement. This statement should provide the following information, where applicable:

- Accession codes, unique identifiers, or web links for publicly available datasets
- A description of any restrictions on data availability
- For clinical datasets or third party data, please ensure that the statement adheres to our policy

A consensus map of ribosomes and maps of related conformations generated in this study were deposited in the EMDB under accession codes EMD-61318 (Consensus map, https://www.ebi.ac.uk/pdbe/entry/emdb/EMD-61318), EMD-61633 (Decoding1 state, https://www.ebi.ac.uk/pdbe/entry/emdb/EMD-61633), EMD-61634 (Decoding2 state, https://www.ebi.ac.uk/pdbe/entry/emdb/EMD-61634). EMD-61635 (DecodingZ state, https://www.ebi.ac.uk/pdbe/entry/emdb/EMD-61635), EMD-61636 (Hibernating state, https://www.ebi.ac.uk/pdbe/entry/emdb/EMD-61636), EMD-61637 (Pre-state with factor, https://www.ebi.ac.uk/pdbe/entry/emdb/EMD-61637), EMD-61638 (Pre-state without factor, https://www.ebi.ac.uk/pdbe/entry/emdb/EMD-61638), EMD-61639 (Rotated1 state, https://www.ebi.ac.uk/pdbe/entry/emdb/EMD-61639), EMD-61640 (Rotated2 state, https://www.ebi.ac.uk/pdbe/entry/emdb/EMD-61640). Raw tomograms generated in this study were deposited in EMPIAR under accession code EMPIAR-12948 (https://www.ebi.ac.uk/empiar/EMPIAR-12948/).

Research involving human participants, their data, or biological material

Policy information about studies with human participants or human data. See also policy information about sex, gender (identity/presentation), and sexual orientation and race, ethnicity and racism.

Reporting on sex and gender	N/A
Reporting on race, ethnicity, or other socially relevant groupings	N/A
Population characteristics	N/A
Recruitment	N/A
Ethics oversight	N/A

Note that full information on the approval of the study protocol must also be provided in the manuscript.

Field-specific reporting

Please select the one below that is the best fit for your research. If you are not sure, read the appropriate sections before making your selection.

☒ Life sciences ☐ Behavioural & social sciences ☐ Ecological, evolutionary & environmental sciences

For a reference copy of the document with all sections, see nature.com/documents/nr-reporting-summary-flat.pdf

Life sciences study design

All studies must disclose on these points even when the disclosure is negative.

Sample size	For voltage imaging, total 568 neurons from 4 grids were tested for voltage imaging.<br>Total 193 tomograms were acquired for structural analysis. The exact numbers and methods of processing the sample was in supplementary Fig 4. The number of tomograms was determined based on available microscope time and number of neurons and ribosomes.
Data exclusions	No data was excluded from the voltage imaging data.<br>Bad tilts of each tilt series and some of tomograms which had contamination or were not well aligned were excluded for clarity of data. From 446231 initial subtomograms were selected by deep learning methods, final 31389 subtomograms were selected for further analysis.
Replication	Replication of voltage imaging was not performed for integrity of neurons. For subtomogram averaging, over 30,000 subtomograms were used for averaging and classification iteratively with different binned subtomograms.
Randomization	Random subtomograms were used for averaging and classification with 60 Å low-pass filter for unbiased processing. Randomized split data set was used to determine resolution at Fourier Shell Correlation 0.143.
Blinding	Because whole neurons on the grids were imaged and used for clustering, blinding was not necessary. Blinding was not performed in Cryo-ET processing because of the unbiased processing, as mentioned above.

Reporting for specific materials, systems and methods

We require information from authors about some types of materials, experimental systems and methods used in many studies. Here, indicate whether each material, system or method listed is relevant to your study. If you are not sure if a list item applies to your research, read the appropriate section before selecting a response.

Materials & experimental systems

n/a	Involved in the study
☒	☐ Antibodies
☐	☒ Eukaryotic cell lines
☒	☐ Palaeontology and archaeology
☐	☒ Animals and other organisms
☒	☐ Clinical data
☒	☐ Dual use research of concern
☒	☐ Plants

Methods

n/a	Involved in the study
☒	☐ ChIP-seq
☒	☐ Flow cytometry
☒	☐ MRI-based neuroimaging

Eukaryotic cell lines

Policy information about cell lines and Sex and Gender in Research

Cell line source(s)	HEK293T(kind gift from Dr. Jongkyeong Chung(Seoul National University)) cells were used for control experiments of voltage imaging.
Authentication	Cell lines were not authenticated
Mycoplasma contamination	Mycoplasma contamination tests were not performed.
Commonly misidentified lines (See ICLAC register)	There are no misidentified lines.

Animals and other research organisms

Policy information about studies involving animals; ARRIVE guidelines recommended for reporting animal research, and Sex and Gender in Research

Laboratory animals	Animals were used only for primary hippocamal cultures. Embryonic day 18 of Rattus norvegicus(Sprague–Dawley) from pregnant female was used.
Wild animals	N/A
Reporting on sex	N/A
Field-collected samples	N/A
Ethics oversight	All animal procedures were approved by the Seoul National University Institutional Animal Care and Use Committee (IACUC # SNU-230703-1).

Note that full information on the approval of the study protocol must also be provided in the manuscript.

Plants

Seed stocks	N/A
Novel plant genotypes	N/A
Authentication	N/A
